# Characterization of dosimetric predictors for pulmonary perfusion changes before and after hypofractionated whole‐breast radiotherapy in node‐negative breast cancer patients: A prospective study

**DOI:** 10.1002/acm2.70641

**Published:** 2026-05-28

**Authors:** Farzaneh Allaveisi, Nima Esmaeilzadeh, Siamak Derakhshan, Smaneh Jamshidi Dizaji, Aian Azadnia

**Affiliations:** ^1^ Department of Medical Physics Faculty of Medicine Kurdistan University of Medical Sciences Sanandaj Iran; ^2^ Department of Student Research Faculty of Medicine Kurdistan University of Medical Sciences Sanandaj Iran; ^3^ Department of Radiotherapy Faculty of Paramedicine Kurdistan University of Medical Sciences Sanandaj Iran; ^4^ Liver and Digestive Research Center Research Institute for Health Development Kurdistan University of Medical Sciences Sanandaj Iran

**Keywords:** breast cancer, dosimetric parameters, hypofractionated radiotherapy, pulmonary function tests, pulmonary perfusion scan, SPECT imaging

## Abstract

**Background:**

Breast cancer is the most common malignancy in women, and adjuvant radiotherapy is an essential component of curative treatment. Despite advances in hypofractionated whole‐breast irradiation (HF‐WBI), incidental lung exposure remains unavoidable and predisposes to radiation‐induced pulmonary injury, including radiation pneumonitis (RP). Traditional predictors such as mean lung dose (MLD) may inadequately capture the heterogeneous intrapulmonary dose distribution typical of breast radiotherapy.

**Purpose:**

The purpose of this study was to evaluate whether heterogeneity‐sensitive dosimetric metrics and functional lung imaging better predict radiation‐induced perfusion changes and RP in patients undergoing HF‐WBI.

**Methods:**

In this prospective study, 48 node‐negative breast cancer patients treated with conformal HF‐WBI were evaluated. Pulmonary perfusion scans (PPS) were acquired pre‐RT and 6–9 months post‐RT. Dose–volume histogram (DVH) parameters, including MLD, uniform effective dose (D_eff_), and V_5_–V_40_, were extracted from three‐dimensional conformal radiotherapy (3DCRT) or forward‐planned intensity‐modulated RT (FIMRT) plans. D_eff_ was calculated using the Lyman–Kutcher–Burman (LKB) model to integrate dose heterogeneity. Associations between dosimetric metrics, perfusion changes, and RP were analyzed using correlation and ROC analysis.

**Results:**

PPS revealed modest but detectable post‐RT perfusion declines, most pronounced in lung regions receiving higher doses. Three patients (6.3%) developed Grade 2 RP. D_eff_ and high‐dose lung volumes (V_35_–V_40_) demonstrated the strongest correlations with perfusion loss and RP (AUC 0.81–0.85, *p* < 0.01), whereas MLD showed weaker predictive value. ROC analysis identified clinically relevant thresholds: D_eff_ ≤ 13.6 Gy, V_35_ ≤ 37.5%, V_40_ ≤ 32%, and a ≥4% decline in total perfusion, below which minimal functional impairment and RP were observed.

**Conclusions:**

D_eff_ and high‐dose volumetric constraints outperform MLD in predicting decrease in lung perfusion in HF‐WBI. Quantitative PPS provides complementary functional validation, and integration of dosimetric and functional parameters may refine lung‐sparing strategies in breast radiotherapy.

## 1 INTRODUCTION

Breast cancer is the most common malignancy among women worldwide. Epidemiological data indicate that approximately one in eight women in the United States will develop breast cancer during their lifetime.^1^, ^2^ Notably, the mean age at diagnosis is lower in several non‐Western regions, including the Middle East, China, and Africa (approximately 48–49 years), compared with Western populations.^3^, ^4^ Advances in screening, systemic therapies, and multidisciplinary management have resulted in substantial improvements in survival, with 5‐year survival rates exceeding 95% in patients with favorable breast cancer subtype.^5^, ^6^, ^7^

As a result, contemporary breast cancer care increasingly emphasizes the reduction of treatment‐related toxicities and the preservation of long‐term quality of life.

Adjuvant radiotherapy, administered after either breast‐conserving surgery or mastectomy, has been shown to substantially decrease the risk of local recurrence and to confer a significant survival benefit.^8^ Hypofractionated whole‐breast irradiation (HF‐WBI) has become the preferred standard of care, as multiple phase III randomized trials have demonstrated its non‐inferiority to conventional fractionation, while offering the additional advantages of reduced treatment duration and improved patient convenience.^9^, ^10^, ^11^ Nevertheless, incidental irradiation of the ipsilateral lung is largely unavoidable, exposing patients to the risk of radiation‐induced pulmonary injury, which ranges from subclinical perfusion abnormalities to clinically apparent radiation pneumonitis (RP).^12^, ^13^, ^14^, ^15^, ^16^, ^17^, ^18^, ^19^, ^20^ The reported incidence of RP varies widely, reflecting differences in radiotherapy techniques, fractionation schedules, and diagnostic criteria.^8^, ^21^, ^22^, ^23^, ^24^, ^25^, ^26^, ^27^, ^28^

A range of dosimetric parameters, including dose–volume histogram (DVH) metrics such as V_5_, V_20_, and V_30_, along with mean lung dose (MLD), have historically been evaluated as potential predictors of radiation‐induced pulmonary toxicity.^18^, ^22^, ^26^, ^28^, ^29^, ^30^, ^31^

The QUANTEC report highlights MLD as a reliable and practical predictor of pulmonary toxicity across various thoracic malignancies.^32^ However, in breast radiotherapy, MLD may inadequately capture the heterogeneous and partial nature of lung irradiation, as similar MLD values can arise from markedly different dose distributions.^33^, ^34^ Evidence suggests that high‐dose exposure of limited lung subvolumes (e.g., V_35_–V_40_) may be more strongly associated with pulmonary toxicity than low‐dose irradiation of larger volumes.^35^ Accordingly, biologically weighted parameters such as the effective dose (D_eff_), derived from the Lyman–Kutcher–Burman (LKB) model, may offer improved predictive performance by integrating dose magnitude and irradiated volume into a single metric.

Beyond conventional dosimetric assessment, functional imaging provides complementary information. Pulmonary perfusion scans (PPS) are capable of detecting radiation‐induced regional perfusion deficits by quantifying vascular alterations secondary to alveolar‐capillary injury. Consequently, PPS may serve as a sensitive biomarker for subclinical radiation‐induced lung injury, potentially preceding or enhancing traditional clinical and radiographic endpoints.^36^, ^37^ However, prospective data correlating PPS‐derived functional changes with detailed dosimetric parameters in breast cancer patients treated with hypofractionated regimens, particularly in the absence of regional nodal irradiation, remain limited.

Therefore, the present prospective study aimed to characterize pulmonary perfusion changes detected by PPS following hypofractionated conformal whole‐breast irradiation in node‐negative breast cancer patients and to evaluate their association with dosimetric parameters, including MLD, effective dose, and high‐dose lung volume indices (V_35_–V_40_). These findings may contribute to improved risk stratification and inform lung‐sparing strategies in contemporary breast radiotherapy.

## 2 MATERIALS AND METHODS

This prospective study was approved by Ethics Committee of the Kurdistan University of Medical Sciences (KUM) and included women newly diagnosed with breast cancer who were referred for adjuvant radiotherapy between January 2020 and November 2021. Additional exclusion criteria comprised current smoking or a prior history of pulmonary disease. Written informed consent was obtained from all participants prior to enrollment.

### 2.1 Patient and treatment characteristics

A total of 48 patients who underwent hypofractionated breast radiotherapy were included in the final analysis. The mean age of the cohort was 46.1 ± 7.8 years, and the mean BMI was 25.2 ± 3.4 kg/m^2^. Tumor laterality was evenly distributed, with 54% of patients presenting with right‐sided and 46% with left‐sided disease. The mean primary tumor size was 2.3 ± 0.8 cm. All patients completed the prescribed radiotherapy course without interruptions. Baseline clinical characteristics and lung dosimetric parameters for the overall cohort are summarized in Table 1.

**TABLE 1:** Baseline characteristics and dosimetric parameters in breast cancer patients (N0) treated with hypofractionated RT using 3D‐CRT or FIMRT.

Variable	Overall cohort (*N* = 48)	3D‐CRT (*N* = 28)	FIMRT (*N* = 20)	*p*‐value
Patient characteristics				
Age (years)	46.1 (7.8)	45.9 (7.6)	46.3 (8.0)	0.78
BMI (kg/m^2^)	25.2 (3.4)	25.1 (3.5)	25.3 (3.3)	0.84
Tumor features				
Tumor location, *n* (%)				0.99
Right breast	54 (54)	27 (54)	27 (54)	
Left breast	46 (46)	23 (46)	23 (46)	
Primary tumor size (cm)	2.3 (0.8)	2.4 (0.9)	2.2 (0.7)	0.39
Dosimetric parameters				
MLD (Gy)	6.8 (1.5)	6.9 (1.4)	6.7 (1.5)	0.47
Deff lung (Gy)	9.2 (2.1)	9.3 (2.0)	9.1 (2.2)	0.58
V5 (%)	32.6 (7.8)	33.2 (7.5)	33.0 (8.1)	0.71
V10 (%)	25.1 (6.7)	25.4 (6.5)	24.8 (6.9)	0.79
V20 (%)	12.4 (4.3)	12.6 (4.2)	12.2 (4.4)	0.82
V30 (%)	7.5 (4.1)	7.6 (4.2)	7.3 (4.0)	0.88
V40 (%)	3.8 (3.2)	3.9 (4.1)	3.2 (3.8)	0.83
Perfusion changes				
Δ Lung perfusion—Upper zone (%)	−1.0 (0.8)	−1.1 (0.8)	−0.9 (0.7)	0.21
Δ Lung perfusion—Middle zone (%)	−2.0 (1.0)	−2.1 (0.9)	−1.9 (1.1)	0.19
Δ Lung perfusion—Lower zone (%)	−1.5 (0.9)	−1.6 (0.8)	−1.4 (0.9)	0.27
Δ Total lung perfusion (%)	−4.5 (2.0)	−4.8 (1.9)	−4.2 (2.1)	0.12

**FIGURE 1:**
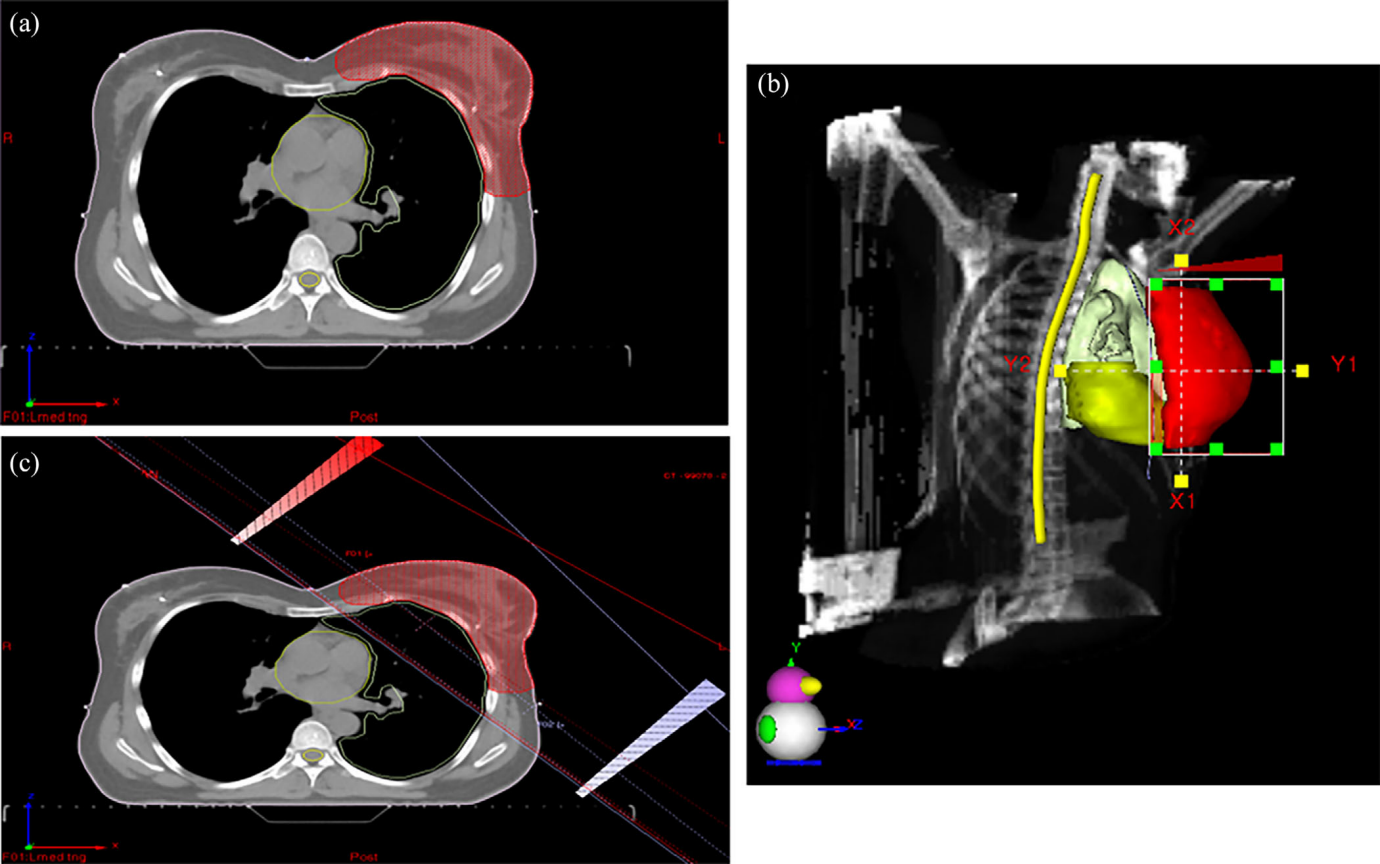
(a) Axial CT slice and (b) digitally reconstructed radiograph (DRR) showing the delineation of the bilateral lung lobes, planning target volume (PTV), and adjacent organs at risk. (c) Treatment fields encompassing medial and lateral tangents.

All patients underwent breast‐conserving surgery and received adjuvant chemotherapy comprising Adriamycin, Cyclophosphamide, and Paclitaxel. The clinical target volume (CTV) was delineated according to RTOG guidelines to encompass the remaining breast tissue.^8^, ^38^ The planning target volume (PTV) was generated by expanding the CTV to account for potential setup uncertainties. Tangential fields were employed, incorporating an anterior flash margin of approximately 1.5–2 cm beyond the breast contour to accommodate respiratory motion and daily patient positioning variations (Figure 1).

A total prescribed dose of 42.56 Gy in 16 fractions was delivered over a three‐week period using tangential three‐dimensional conformal radiotherapy (3DCRT) fields. Dose homogeneity was optimized through the use of either wedge filters or forward‐planned intensity‐modulated radiotherapy (FIMRT) techniques. Organs at risk (OARs), including the ipsilateral lung and heart, were carefully contoured and evaluated on planning CT scans to ensure compliance with established dose constraints (Figure 1).

Dosimetric parameters, including MLD, D_eff_, and the percentage of ipsilateral lung volume receiving doses between 5 and 40 Gy (V_5_–V_40_), were extracted from dose–volume histograms (DVHs).

Pulmonary perfusion scans were performed both prior to radiotherapy and at 6–9 months post‐treatment (mean: 7.5 months). PPS acquisition involved intravenous administration of 185 MBq [99mTc] Tc‐macroaggregated albumin (MAA) in the supine position, followed by planar imaging (1000 k counts per view, 256 × 256 matrix) and SPECT acquisition (64 views over 360°, 15 s per view, 64 × 64 matrix) using a dual‐head ECAM Scintron gamma camera (MiE Medical Imaging Electronics, Hamburg, Germany) equipped with a low‐energy high‐resolution collimator. SPECT images were reconstructed using ordered subsets expectation maximization (OSEM) with eight iterations and four subsets.

Relative perfusion in the upper, middle, and lower lung zones was automatically quantified using Scintron7 software from anterior and posterior planar views, with the geometric mean of both views employed for quantitative analysis. All PPS images were independently reviewed by a board‐certified nuclear medicine physician who was blinded to patient treatment information.

### 2.2 Equivalent Uniform Dose (Effective Dose)

To account for spatial heterogeneity in intrapulmonary dose distribution, the Kutcher equivalent uniform dose (EUD_Kutcher_) was calculated for each patient using DVH data extracted from the corresponding 3DCRT treatment plans. EUD_Kutcher_ represents a biologically weighted average dose that integrates both dose magnitude and volumetric effects, thereby providing a physiologically more relevant parameter than the arithmetic mean dose. This metric is widely employed in normal tissue complication probability (NTCP) modeling to estimate the risk of radiation‐induced functional impairment.

EUD_Kutcher_ ​ was calculated according to the LKB model,^39^, ^40^ expressed as:

$$\mathrm{EUD}={\left(\sum_{i}v_{i}D_{i}^{a}\right)}^{\frac{1}{a}}$$

where *D_i_* is the dose to the differential volume element *v_i_* ​, and *a* =$\;\frac{1}{n}$, with n denoting the volume‐effect parameter that reflects tissue sensitivity to dose heterogeneity. For lung tissue, we adopted an n value of 0.53, as reported by Rancati et al.,^41^ for the endpoint of symptomatic radiation pneumonitis in combination with radiologically assessed lung density changes. This selection is consistent with the established understanding that even partial lung irradiation may lead to clinically meaningful functional impairment.

EUD_Kutcher_ ​ values were computed for all patients and subsequently analyzed with respect to their association with radiation pneumonitis and post‐treatment reductions in lung volume. In exploratory analyses, potential threshold levels of EUD_Kutcher_ ​ were identified, below which no cases of clinically significant RP or substantial perfusion loss were observed.

### 2.3 Statistical analysis

Continuous variables were summarized as median (range) and categorical variables as frequency (percentage). Fisher's exact test, Student's *t*‐test, and Mann–Whitney *U* test were applied to compare PPS measurements and dosimetric parameters (MLD, D_eff_, V_5_–V_40_) between pre‐ and post‐radiotherapy assessments. Correlations between dosimetric parameters and PPS changes were evaluated using Spearman's rank correlation coefficient. The Shapiro–Wilk test was used to assess PPS data normality, with non‐normally distributed values log‐transformed prior to analysis.

DVH parameters for the ipsilateral lung (MLD, D_eff_, V_5_–V_40_) were analyzed for their association with post‐treatment PPS changes. Multivariable linear and logistic regression models, adjusting for age (continuous), body mass index (continuous), surgery type (breast‐conserving vs. modified radical mastectomy), chemotherapy regimen, and tumor laterality (left vs. right), were employed to determine the independent effect of dosimetric parameters on PPS reduction. For parameters significantly associated with PPS reduction, receiver operating characteristic (ROC) curve analysis was performed to identify optimal cutoff values, giving equal weight to sensitivity and specificity.

All statistical analyses were conducted using R software version 3.6.1 (The R Foundation for Statistical Computing, Vienna, Austria; http://www.r‐project.org). A two‐sided *p*‐value of <0.05 was considered statistically significant.

## 3 RESULTS

### 3.1 Incidence of radiation pneumonitis

During follow‐up, three patients (6.3%) developed symptomatic Grade 2 radiation pneumonitis. Given the small number of events, statistical modeling of RP incidence was not feasible. Therefore, the analysis focuses on lung dosimetric parameters and regional perfusion changes rather than RP frequency.

### 3.2 Quantitative evaluation of regional pulmonary perfusion alterations

**FIGURE 2:**
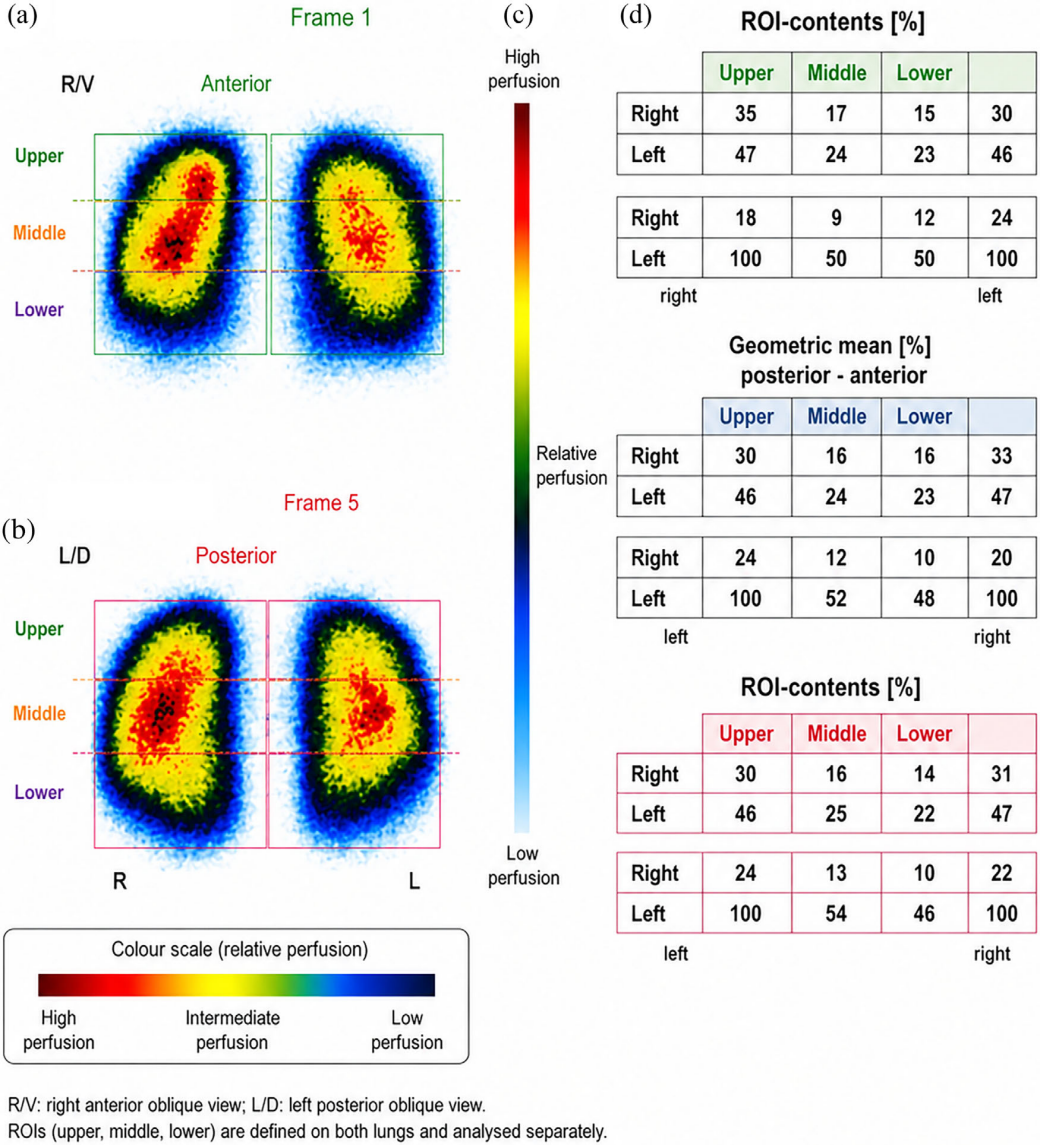
Automated workflow for regional pulmonary perfusion analysis based on anterior and posterior planar scintigraphy. (a) Representative anterior (top) and posterior (bottom) lung perfusion images with superimposed regions of interest (ROIs), in which each lung is subdivided into anatomically defined upper, middle, and lower zones. (b) Corresponding quantitative perfusion values (%) obtained from each ROI for both projections, together with the calculated geometric mean values for each lung region. (c) Color scale representing relative perfusion intensity, where red denotes high perfusion, yellow indicates intermediate perfusion, and blue reflects low perfusion. (d) Schematic overview of the analysis pipeline: anterior and posterior images are segmented into upper, middle, and lower ROIs; regional perfusion values are extracted for each projection; and geometric mean values are computed to provide a projection‐independent estimate of regional pulmonary perfusion.

**FIGURE 3:**
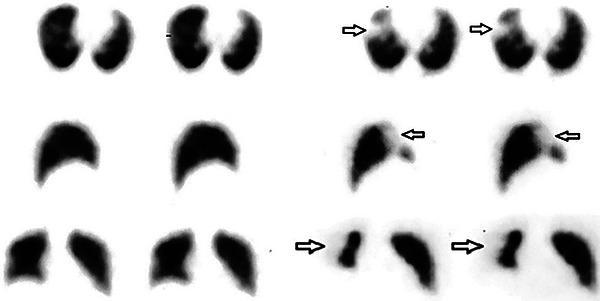
Transverse (top), sagittal (middle), and coronal (bottom) SPECT lung images obtained prior to (left) and following (right) radiotherapy for right‐sided breast cancer. Post‐treatment images reveal distinct perfusion defects localized to the middle and upper regions of the right lung (arrows).

**FIGURE 4:**
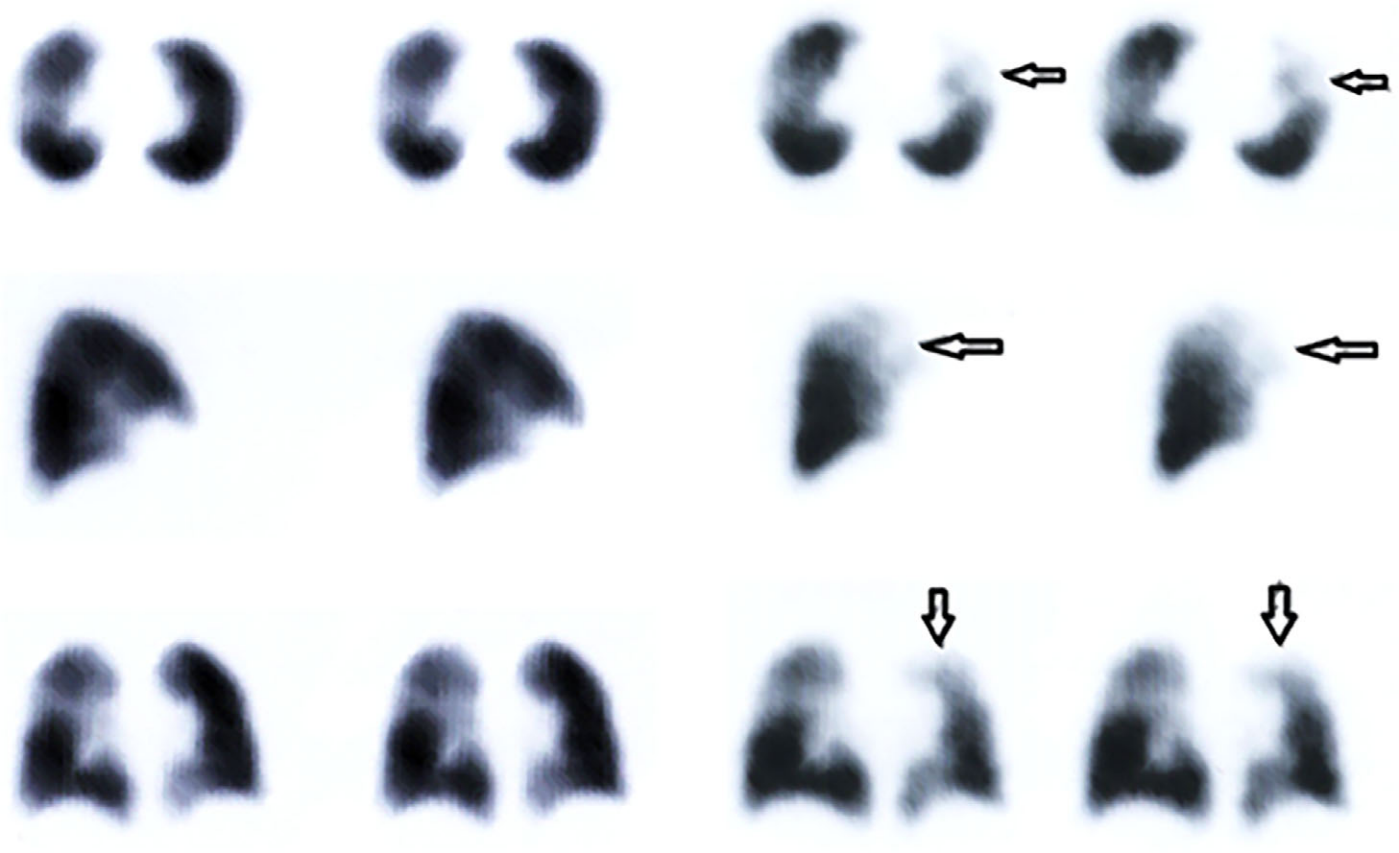
Pre‐ (left) and post‐radiotherapy (right) transverse (top), sagittal (middle), and coronal (bottom) SPECT images of the lungs in a patient with left breast cancer. The post‐treatment scans reveal perfusion defects involving the middle and upper lobes of the left lung (arrows).

Quantitative assessment of pulmonary perfusion following hypofractionated breast radiotherapy revealed modest but detectable reductions across all lung regions. The mean decreases in relative perfusion were –1.0%, –2.0%, and –1.5% in the upper, middle, and lower lung zones, respectively, resulting in an overall decline of –4.5% in total lung perfusion (Table 1, Figures 2, 3, 4).

**FIGURE 5:**
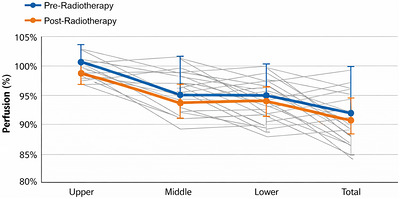
Changes in regional pulmonary perfusion before and after hypofractionated breast radiotherapy. Mean perfusion values before (blue) and after (orange) hypofractionated whole‐breast radiotherapy, with individual patient data shown as grey lines and group means ± standard deviations shown as blue (pre) and orange (post) lines.

Pulmonary perfusion measurements before and after radiotherapy are presented in Figure 5, illustrating both individual patient trajectories and mean reductions across the upper, middle, lower, and total lung regions. While the majority of patients exhibited a post‐treatment decline in perfusion, a subset showed minimal or no change, reflecting inter‐patient variability.

Comparative analysis between the 3D‐CRT and FIMRT cohorts showed no statistically significant differences in perfusion changes (*p* > 0.1).

### 3.3 Dosimetric parameters

The mean values of the dosimetric parameters of the lungs are summarized in Table 1. The mean MLD and D_eff_ were 6.8 Gy and 9.2 Gy, respectively. The mean V_5_, V_10_, V_20_, V_30_, and V_40_ of the ipsilateral lung were 32.6%, 25.1%, 12.4%, 7.5%, and 3.8%, respectively. No statistically significant differences were observed between the 3D‐CRT and FIMRT techniques for any of these parameters.

### 3.4 Correlation between dosimetric parameters and changes in relative lung perfusion

Spearman correlation analysis demonstrated significant negative correlations between changes in relative lung perfusion and multiple dose–volume parameters, including V_20_ (*r* = –0.42, *p* = 0.015), V_25_ (*r* = –0.48, *p* = 0.008), V_30_ (*r* = –0.51, *p* = 0.005), V_35_ (*r* = –0.59, *p* < 0.001), and V_40_ (*r* = –0.62, *p* < 0.001). Both MLD (*r* = –0.46, *p* = 0.010) and D_eff_ (*r* = –0.57, *p* = 0.002) showed significant correlations, with D_eff_ demonstrating the highest correlation coefficient. These findings suggest that higher doses to even moderately large lung volumes are strongly associated with functional impairment, and that D_eff_ may be the most sensitive dosimetric predictor among those tested.

Importantly, reductions in pulmonary perfusion at six months post‐treatment reflect subclinical radiation‐induced lung injury rather than serving as predictors of clinical RP.

### 3.5 ROC curve analysis and optimal cut‐off values

ROC curve analysis demonstrated that the most predictive parameters for clinically relevant perfusion decline were V_35_, V_40_, and D_eff_. Specifically, V_35_ exhibited an AUC of 0.81 with an optimal cut‐off of 37.5%, V_40_ had an AUC of 0.85 with a cut‐off of 32.0%, and D_eff_ showed an AUC of 0.83 with a cut‐off of 13.6 Gy. These thresholds indicate that higher V_35_, V_40_, and D_eff_ values are associated with greater reductions in regional and total lung perfusion, providing potential clinical benchmarks to guide treatment planning and minimize functional impairment following hypofractionated breast radiotherapy.

## 4 DISCUSSION

In this prospective study of node‐negative breast cancer patients treated with hypofractionated WBI using either 3D‐CRT or FIMRT, we assessed the incidence of radiation pneumonitis and identified dosimetric and functional predictors of pulmonary toxicity. Baseline clinical and anatomical characteristics were well balanced between the two groups (Table 1). Notably, lung dose distributions were comparable across 3D‐CRT and FIMRT cohorts, and the incidence of RP (three patients, 6.3%) did not differ significantly by technique, highlighting that overall dose–volume exposure, rather than delivery modality, predominantly determines the risk of pulmonary toxicity.

Among conventional dosimetric metrics and the effective dose derived from the Lyman‐Kutcher‐Burman (LKB) model (D_eff_), along with high‐dose lung volumes (V_35_–V_40_ **),** Deff and V_35_–V_40_ emerged as the most reliable predictors of Grade 2 radiation pneumonitis (RP). D_eff_ exhibited superior discriminative performance, reflecting both its independence from lung size and its biological relevance in the context of parallel‐organ architecture.^41^, ^42^, ^43^, ^44^ In contrast, MLD proved to be less robust, as it is influenced by total lung volume and may underestimate the risk of pulmonary toxicity in certain patients. This observation is consistent with prior studies in thoracic oncology, which suggest that D_eff_ offers a more clinically meaningful estimate of lung injury than MLD.^33^, ^34^

ROC analysis further identified clinically relevant thresholds, including D_eff_ > 13.6 Gy, V_35_ > 37.5%, V_40_ > 32%, and a ≥ 4% decline in total lung perfusion, which were associated with patterns of radiation‐induced pulmonary changes.

Intermediate‐ to high‐dose volumetric parameters (V_30_–V_35_) also demonstrated strong correlations. These findings suggest that even small lung subvolumes receiving ≥30–40 Gy were more prominently associated with regions showing greater perfusion reduction following radiotherapy, indicating potential spatial heterogeneity in radiation‐induced pulmonary effects. In line with the principle that “a large dose to a small volume” is more detrimental than “a small dose to a large volume”.^27^, ^33^, ^34^, ^35^

A novel aspect of this study is the integration of functional imaging. Quantitative perfusion SPECT revealed that a ≥4% decline in total lung perfusion reflects subclinical radiation‐induced lung injury rather than predicting clinical RP. The concordance between functional perfusion declines and clinical RP at comparable dosimetric thresholds supports the biological validity of these metrics and underscores the potential of functional dosimetry to enhance risk stratification.^12^, ^29^, ^31^, ^44^, ^45^, ^46^

Collectively, these findings underscore the limitations of relying solely on MLD. An integrative approach that combines D_eff_, high‐dose volumetric constraints (V_35_–V_40_), and functional perfusion changes may provide a more accurate and individualized predictive model. Clinically, this framework could guide patient selection for lung‐sparing strategies, such as deep inspiration breath‐hold, prone positioning, or partial tangents, particularly in patients with unfavorable anatomy or borderline dose constraints.^8^, ^22^

While this study focused on a mildly hypofractionated regimen of 42.56 Gy in 16 fractions (Canadian fractionation), it is important to note that conventional fractionation (50 Gy in 25 fractions) has been widely used and evaluated in breast radiotherapy. Multiple randomized trials, including the Canadian trial and the START A/B trials, have demonstrated comparable local control, overall survival, and toxicity profiles between these regimens.^9^, ^10^, ^20^, ^47^ The Canadian hypofractionation schedule offers the advantages of reduced treatment duration and patient convenience, while maintaining non‐inferior outcomes. Although the present study did not directly compare these fractionation schemes, existing literature supports the clinical equivalence of these approaches regarding both tumor control and radiation‐induced pulmonary effects.

In addition to dosimetric factors, systemic therapies may influence the risk of radiation‐induced lung injury. Previous studies have suggested that hormonal agents, such as tamoxifen, as well as certain chemotherapeutic drugs, may enhance pulmonary radiosensitivity through inflammatory or fibrotic pathways. Although patients in the present cohort received standard adjuvant systemic treatments, the study was not designed to assess the independent contribution of specific medications to pulmonary toxicity. This represents a limitation of the current analysis and should be addressed in future studies with larger cohorts and detailed stratification of systemic therapies.

This study has several limitations. RP grading was performed according to standard clinical criteria, with Grade 2 RP defined as moderately symptomatic rather than subclinical.

Treatment planning was conducted using free‐breathing CT scans without respiratory motion management, and nodal irradiation was not included, potentially limiting the generalizability of the findings. Finally, the sample size was modest, and external validation of the proposed dosimetric and functional thresholds is warranted.

## 5 CONCLUSION

In hypofractionated whole‐breast radiotherapy for node‐negative patients, D_eff_ and high‐dose volumetric metrics (V_35_–V_40_) outperform MLD in predicting radiation pneumonitis. Quantitative perfusion decline on SPECT provides complementary functional information, with a ≥4% reduction in total lung perfusion serving as a strong predictor of decrease in lung perfusion. Practical thresholds derived from this study, D_eff_ < 13.6 Gy, V_35_ < 37.5%, V_40_ < 32%, may be applied as planning constraints to minimize pulmonary toxicity while preserving target coverage.

## AUTHOR CONTRIBUTIONS

Farzaneh Allaveisi was involved in the study design, data analysis, interpretation of the findings, and preparation of the manuscript. Nima Esmaeilzadeh participated in data collection. Siamak Derakhshan and Samaneh Jamshidi Dizaji contributed to patient recruitment and clinical data acquisition. Arain Azadnia performed the statistical analysis and assisted with interpretation of the results. All authors read and approved the final version of the manuscript.

## CONFLICT OF INTEREST STATEMENT

The authors declare that they have no known competing financial interests or personal relationships that could have appeared to influence the work reported in this paper.

## ETHICAL STATEMENT

This prospective study was approved by the Institutional Ethics Committee of Kurdistan University of Medical Sciences (KUM) (approval code: IR.MUK.REC.1396/305) and included women newly diagnosed with breast cancer who were referred for adjuvant radiotherapy between January 2020 and November 2021. Written informed consent was obtained from all participants prior to enrollment.

## Data Availability

The datasets generated and analyzed during the current study are available from the corresponding author on reasonable request.
